# The cotyloid fossa fat pad as a hip-specific orthobiologic niche: lessons from the infrapatellar fat pad for mesenchymal stem/stromal cell and extracellular vesicle therapy in osteoarthritis

**DOI:** 10.3389/fbioe.2026.1930127

**Published:** 2026-10-28

**Authors:** R. P. Rossow, K. Polavarapu, M. Naik, L. D. Kaplan, T. M. Best, M. G. Rizzo, D. Kouroupis

**Affiliations:** 1 Department of Orthopaedics, UHealth Sports Medicine Institute, Miller School of Medicine, University of Miami, Miami, FL, United States; 2 Diabetes Research Institute and Cell Transplant Center, Miller School of Medicine, University of Miami, Miami, FL, United States

**Keywords:** mesenchymal stem/stromal cells, osteoarthritis, extracellular vesicles, infrapatellar fat pad, cotyloid fossa fat pad, immunomodulation, orthobiologics

## Abstract

Hip osteoarthritis (OA) is a leading cause of pain and disability worldwide, yet no validated disease-modifying orthobiologic therapy currently exists for the hip joint. The infrapatellar fat pad (IFP) has emerged as a well-characterized intra-articular adipose-synovial niche in the knee, serving as an accessible source of mesenchymal stem/stromal cells (MSC) with robust immunomodulatory, anti-fibrotic, and chondroprotective properties. IFP-MSC have been extensively characterized through surface immunophenotyping, clonogenic assays, secretome profiling, and multilineage differentiation studies, and their therapeutic capacity has been substantially enhanced through cytokine-based, hypoxic, and hormonal priming paradigms. Their derived extracellular vesicles (EV) further extend this therapeutic framework through paracrine immunomodulation, macrophage polarization, and engineered cargo delivery. The cotyloid fossa fat pad (CFFP), an anatomically analogous adipose-synovial structure within the acetabular fossa of the hip, has received comparatively little biological characterization despite its proximity to the acetabular cartilage, labrum, and ligamentum teres. In this review, we use the well-characterized IFP/synovium complex as a reference framework to systematically evaluate what is established and what remains undefined about the CFFP/pulvinar region in the context of hip OA pathogenesis and cell-based therapy. We synthesize evidence across joint pathology, cellular and molecular composition, synovial interactions, immunometabolic phenotype, and translational readiness. Our review revealed a consistent asymmetry in which the IFP reflects an established and growing evidence base while the CFFP has received limited but emerging direct characterization, with its MSC potency, priming responses, and EV function still undefined. We conclude that the IFP/CFFP comparison is scientifically testable and clinically compelling, but that CFFP-MSC and CFFP-EV cannot yet be presented as validated hip-specific orthobiologic candidates until foundational characterization and head-to-head functional comparisons have been completed.

## 1 Introduction

The cotyloid fossa is a hollow space located at the center of the acetabulum composed of adipose and synovial tissue. This region envelops the ligamentum teres and contributes to hip joint stability, lubrication, and the distribution of mechanical loads during motion (Standring, 2020; Slullitel et al., 2020). Once regarded as a passive space-filling structure, the cotyloid fossa is now recognized as an active participant in hip joint physiology (Slullitel et al., 2020; Tilouche et al., 2026). The cotyloid fossa synovium houses a vascularized stromal compartment with resident immune cells and mesenchymal progenitors that actively maintain joint homeostasis and respond to mechanical stress and tissue injury (Slullitel et al., 2020; Tilouche et al., 2026; Blackler et al., 2025; Murata et al., 2018). Recent investigations have identified mesenchymal stem/stromal cells (MSC) locally that are capable of osteogenic and chondrogenic differentiation; suggesting that this tissue may contribute to joint maintenance and repair (Tilouche et al., 2026; Murata et al., 2018; McGonagle et al., 2017). However, in conditions such as hip osteoarthritis (OA), the cotyloid fossa region shows inflammatory and remodeling changes, characterized by increased pro-inflammatory mediator and matrix-degrading enzyme expression that may contribute to cartilage degeneration and joint pain (Slullitel et al., 2020; Tilouche et al., 2026; Kuhns et al., 2025; Eymard et al., 2017).

A comparable intra-articular adipose-synovial structure in the knee is the infrapatellar fat pad (IFP), which has been extensively studied as an immunometabolic niche and MSC source in knee OA (Eymard et al., 2017; Greif et al., 2020). Both the cotyloid fossa fat pad (CFFP) and the IFP operate as integrated fat-synovial complexes that regulate local inflammatory signaling and tissue remodeling within their respective joints (Slullitel et al., 2020; Tilouche et al., 2026; Eymard et al., 2017; Greif et al., 2020; Robinson et al., 2016). A direct comparison of these tissues would clarify whether joint-specific adipose-synovial niches contribute differently to OA initiation, inflammatory progression, and biological repair.

Herein, we use the well-characterized IFP/synovium complex as a reference model to evaluate whether the cotyloid fossa/pulvinar region may represent a hip-specific counterpart. We detail the established evidence, identify gaps in CFFP-MSC and CFFP-EV biology, and outline the studies needed to test whether hip-resident MSC/EV products offer joint-specific therapeutic advantages. Extensive work has established that IFP-MSC possess robust immunomodulatory and anti-fibrotic properties, including CD10-mediated Substance P (SP) degradation, modulation of Calcitonin Gene-Related Peptide (CGRP)-associated neurogenic inflammation, and enhanced therapeutic efficacy through inflammatory priming and extracellular vesicle (EV) engineering (Greif et al., 2020; Ioan-Facsinay and Kloppenburg, 2013; Kouroupis et al., 2019; Liebmann et al., 2025). These approaches have demonstrated the capacity to attenuate synovitis, reverse fat pad fibrosis, and reduce pain-related signaling in preclinical and early translational models. We adopt the IFP/synovium as the reference model because it is the most rigorously characterized intra-articular adipose-synovial MSC niche. Specifically, it shares the cotyloid fossa’s defining features, including intra-articular fibrofatty structure, synovial integration, a vascularized stromal compartment, and resident MSC under cyclic joint loading, while we recognize the hip-specific anatomic and mechanical differences.

In contrast, the cellular phenotype, immunometabolic profile, priming responsiveness, and EV functional capacity of CFFP-MSC remain largely undefined. No studies have directly compared CFFP-MSC to IFP-MSC under inflammatory conditions or tested whether they support CD10 enrichment, neuropeptide modulation, or targeted EV-based engineering. We therefore contrast the well-characterized IFP therapeutic paradigm with the limited but emerging literature on the CFFP/synovium region in hip OA. Finally, we outline a translational roadmap to determine whether hip-resident MSC/EV offer joint-specific advantages for disease- and pain-modifying OA therapy.

This narrative review synthesized literature identified through PubMed/MEDLINE, Embase, and Scopus searches, using combinations of the terms infrapatellar fat pad, cotyloid fossa, pulvinar, acetabular fossa, mesenchymal stem/stromal cell, extracellular vesicle, osteoarthritis, synovium, and immunomodulation. English-language peer-reviewed articles were included. As a narrative review, no formal risk-of-bias instrument was applied; studies were selected for relevance and appraised qualitatively.

## 2 Immune-related joint pathology

### 2.1 Pathogenesis of OA in knee and hip joints

OA is the most common joint disease, and develops from an interplay of aging, mechanical stress, metabolic factors, and chronic inflammation (Robinson et al., 2016; Katz et al., 2021; Klop et al., 2025; Loeser, 2009). Primary OA typically appears gradually with age and repetitive loading, while secondary OA follows injury, congenital abnormalities, or metabolic disorders (Katz et al., 2021; Klop et al., 2025; Lespasio et al., 2018). Regardless of where it originates, both forms share common pathogenic mechanisms involving progressive disruption of cartilage homeostasis and persistent low-grade inflammation (Robinson et al., 2016; Kapoor et al., 2011). Chondrocytes progressively lose their ability to maintain extracellular matrix integrity and instead adopt a catabolic phenotype characterized by production of inflammatory mediators, matrix-degrading enzymes, and senescence-associated secretory factors associated with aging (Robinson et al., 2016; Loeser, 2009). These early molecular changes contribute to a joint microenvironment that favors tissue breakdown rather than repair (Robinson et al., 2016; Loeser, 2009; Kapoor et al., 2011).

A consistent finding in OA research is that synovial inflammation is often present even in early disease, well before measurable cartilage loss occurs. Activated synovium produces inflammatory cytokines, including IL-1β, TNF-α, and IL-6, that impair cartilage homeostasis (Zeng et al., 2020; Bondeson et al., 2006; Scanzello and Goldring, 2012). These mediators upregulate matrix-degrading enzymes including MMP-13 and ADAMTS-5 while simultaneously suppressing the anabolic gene programs required for cartilage repair (Scanzello and Goldring, 2012; Stöckl et al., 2021; Zhang et al., 2020). As the cartilage degrades, extracellular matrix fragments are released into the joint space, further activating synovial immune cells and perpetuating inflammation that establishes a cycle in which structural damage and inflammatory signaling amplify one another.

In the knee, one of the most important contributors to this cycle is the IFP. Historically viewed as a passive tissue, the IFP is now recognized as an active immunological and metabolic organ containing macrophages, fibroblasts, adipocytes, and resident MSC that respond rapidly to joint stress (Greif et al., 2020; Liu et al., 2019). During OA, IFP macrophages shift toward an M1-like pro-inflammatory state; then, release cytokines, prostaglandins, and adipokines such as IL-6, TNF-α, and leptin (Greif et al., 2020; Ioan-Facsinay and Kloppenburg, 2013). These mediators correlate with cartilage thinning and patient-reported symptoms (Stannus et al., 2010; Stannus et al., 2013; Gao et al., 2020). As inflammation persists, the IFP becomes fibrotic with increased vascularization, resulting in increased tissue stiffness that increases mechanical stress on the knee- making it both a sensor and amplifier of disease progression (Greif et al., 2020; Zeng et al., 2020; Clockaerts et al., 2010; Emmi et al., 2022; Okada et al., 2025).

In the hip, analogous mechanisms are plausible in the cotyloid fossa/pulvinar region, but the evidence base remains much thinner than for the IFP. Kuhns et al. documented inflammatory and remodeling changes in pulvinar/cotyloid fossa synovial tissue through biochemical and histologic analysis of FAI patients, though direct CFFP-MSC and CFFP-EV characterization remains limited (Tilouche et al., 2026; Murata et al., 2018; Kuhns et al., 2025). Given the cotyloid fossa’s proximity to the acetabular cartilage, an emerging theory is that host inflammation may contribute to cartilage wear, labral degeneration, and pain (Slullitel et al., 2020; Kuhns et al., 2025; Trisolino et al., 2020).

Altogether, OA is not just a disease of articular cartilage. It is a condition involving continuous communication among cartilage, synovium, bone, adipose tissue, immune cells, and resident MSC. The IFP and the CFFP are central hubs in this network. Understanding how each contributes to inflammation and how their MSC populations respond to disease is a prerequisite for developing joint-specific biological therapies for knee and hip OA. Figure 1 illustrates this proposed shared pathogenic framework, depicting how mechanical stress and inflammatory cytokines activate the IFP in the knee and, by biological analogy, are hypothesized to trigger analogous responses in the CFFP of the hip.

**FIGURE 1:**
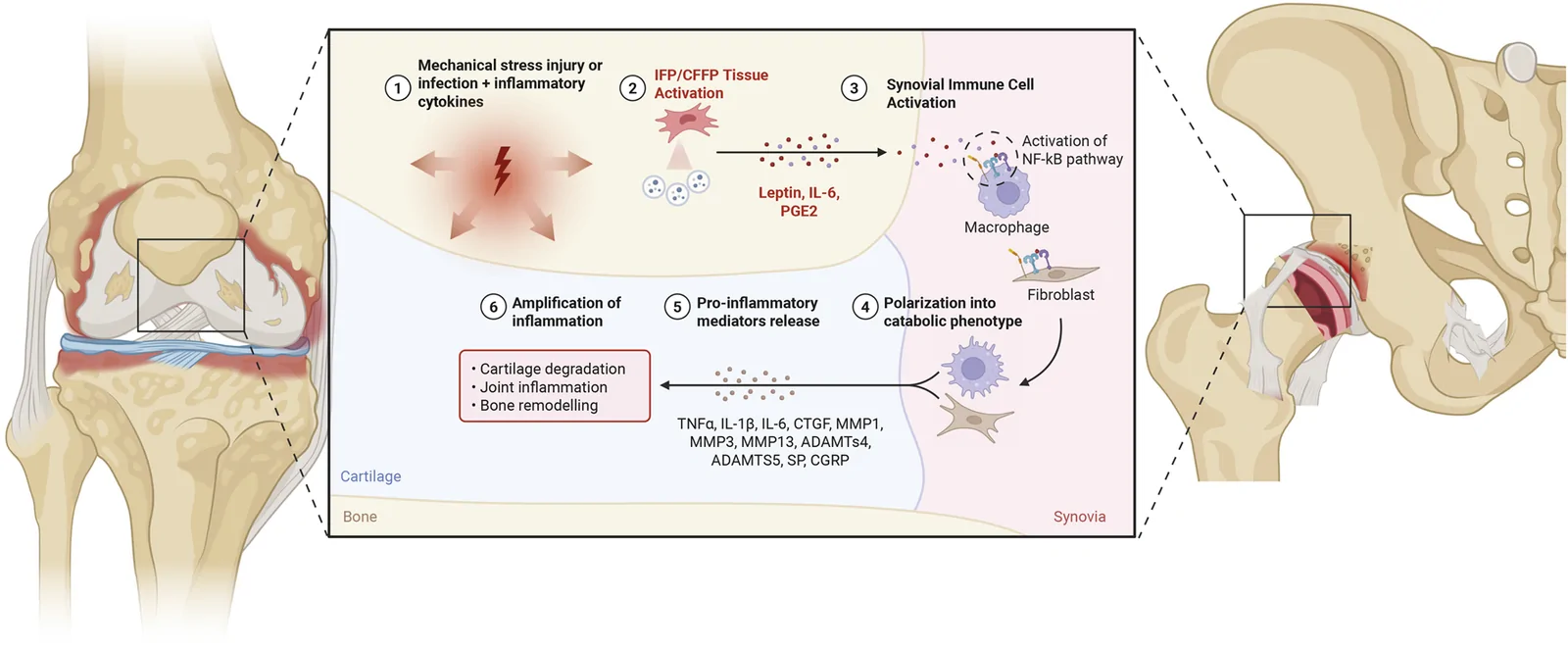
Proposed shared pathogenic mechanisms of knee and hip OA involving IFP activation and hypothesized CFFP/pulvinar tissue activation. (1) Mechanical stress, injury, or inflammatory cytokines (2) trigger activation of the IFP and, by hypothesis, the CFFP/pulvinar region, releasing DAMPs and adipokines including leptin, IL-6, and PGE2. (3) These signals drive synovial immune cell activation via the NF-κB pathway in macrophages and fibroblasts. (4) This induces polarization toward a catabolic phenotype, (5) which results in the release of pro-inflammatory mediators (TNFα, IL-1β, IL-6, Connective tissue growth factor (CTGF), MMP-1, MMP-3, MMP-13, ADAMTS-4, ADAMTS-5, SP, and CGRP. (6) The consequence of this cascade is joint-wide inflammation, cartilage degradation, and bone remodeling in a self-amplifying cycle. Created in BioRender. Rossow, R. (2026).

### 2.2 Immune infiltration and clinical correlates in joint disease

OA is increasingly understood not as an isolated cartilage disorder, but as a disease of the joint as an integrated organ. The evidence base differs substantially by joint: the IFP has been studied through histology, transcriptomics, imaging, and functional MSC assays, whereas the cotyloid fossa/pulvinar literature is still dominated by observational, anatomic, and synovial tissue studies. Synovium, subchondral bone, periarticular fat pads, immune cells, and nociceptive pathways interact to drive inflammation, tissue remodeling, and pain sensitization (Robinson et al., 2016; Klop et al., 2025; Dragoo et al., 2012). Synovitis plays a central etiological role in OA progression, both by perpetuating local inflammatory cascades and by sensitizing joint nociceptors, thereby linking structural degeneration to clinical symptoms (Robinson et al., 2016; Kouroupis et al., 2021; Mathiessen and Conaghan, 2017). Within this framework, the IFP functions as a critical immunometabolic node, with adipocyte- and immune cell-derived cytokines, adipokines, and neurogenic mediators driving synovial hyperplasia, fibrosis, and persistent inflammation (Greif et al., 2020; Zeng et al., 2020; Bastiaansen-Jenniskens et al., 2013).

The role of the IFP in knee OA provides a clear example of how fat pad-driven inflammation integrates mechanical stress and immune dysregulation in the pathogenesis of OA. IFP fibrosis, collagen deposition, and neurogenic inflammation correlate with altered muscle coordination, reduced pain thresholds, and anterior knee pain, highlighting a direct link between fat pad pathology and central pain processing (Zeng et al., 2020; Dragoo et al., 2012; Belluzzi et al., 2019; Hodges et al., 2009). Conditions such as Hoffa’s fat pad impingement further demonstrate the pathological relevance of this tissue: mechanical compression of a densely innervated, inflamed structure exacerbates nociceptive signaling and functional limitation (Ioan-Facsinay and Kloppenburg, 2013; Dragoo et al., 2012).

Other neurogenic mediators produced within the IFP that have been found to drive neurogenic inflammation, pain transmission, and immune modulation in OA are SP and CGRP (Liebmann et al., 2025; Liebmann et al., 2024). SP promotes inflammatory responses and nociceptive signaling, while CGRP induces apoptosis, senescence, and reduces chondrogenic marker expression in OA chondrocytes (Kouroupis et al., 2019; Stöckl et al., 2021). IFP-MSC target neurogenic inflammation by upregulating CD10/neprilysin in response to pro-inflammatory and pro-fibrotic cues, enabling active degradation of SP both *in vitro* and *in vivo* (Kouroupis et al., 2019; Kouroupis et al., 2020a). This SP degradation capacity has been demonstrated to reverse synovitis and fat pad fibrosis in preclinical model (Kouroupis et al., 2019; Kouroupis et al., 2021; Kouroupis et al., 2020a).

In contrast, hip pathology has traditionally been framed through a predominantly mechanical lens, with the CFFP largely regarded as a structural element. In hip OA, inflammation of the cotyloid fossa synovium correlates with labral pathology and functional disability (Trisolino et al., 2020). However, this does not appear to scale directly with fat pad size or gross morphologic parameters, suggesting that inflammatory signaling at this site may arise from mechanical insult or secondary synovial activation rather than constitutive metabolic activity (Slullitel et al., 2020; Murata et al., 2022). This distinction has contributed to the prevailing view of the CFFP as less biologically active than the IFP, a conclusion that may reflect an evidence gap rather than a true biological difference.

When these observations are viewed within the broader OA paradigm, a unifying hypothesis emerges. Periarticular fat pads act as context-dependent regulators of joint inflammation, with their pathogenic contribution shaped by joint biomechanics, immune infiltration, and synovial crosstalk. In the knee, the IFP clearly functions as an inflammatory amplifier and biomarker reservoir (Ioan-Facsinay and Kloppenburg, 2013; Zeng et al., 2020; Wijesinghe et al., 2026). In the hip, while current data link the cotyloid fossa more strongly to structural pathology, the presence of synovitis, immune infiltration, and clinical correlates of disability suggests that similar fat-synovium-immune interactions may exist but remain underexplored (Kuhns et al., 2025; Eymard et al., 2017; Robinson et al., 2016; Murata et al., 2022). The key structural, pathological, and molecular distinctions between these two tissues are summarized in Table 1. Notably, while the IFP is well-characterized as an active inflammatory driver with defined neurogenic mediators and clinical correlates, the CFFP column reflects a consistent pattern of biological plausibility without direct characterization.

**TABLE 1:** Comparative features of the IFP and CFFP.

Feature	IFP (knee)	CFFP (hip; pulvinar/cotyloid fossa synovium)
Structure name	Hoffa’s fat pad	Cotyloid fossa fat pad (pulvinar)
Associated pathologies	Knee OA; Hoffa’s fat pad impingement syndrome	FAI-associated synovitis; hip OA
Immune/molecular role	Well-characterized inflammatory driver; elevated IL-6, pro-inflammatory cytokines, and adipokines. Neurogenic mediators (SP, CGRP) promote synovitis, fibrosis, and pain and are targeted by CD10^+^ IFP-MSC immunomodulation	Emerging evidence of synovial inflammation in the pulvinar/cotyloid fossa region. Direct inflammatory mediator profiling of the CFFP remains limited; its role as a primary inflammatory driver is not yet established
Key clinical findings	Associated with anterior knee pain, altered PPTs, and neuromuscular dysfunction; contributes to pain sensitization and joint pathology	Cotyloid fossa dimensions (CFW/CFH) correlate with labral tear size and functional disability (SF-12); synovitis observed in early hip OA

## 3 Cellular composition and molecular mediators

### 3.1 IFP and CFFP cellular composition

Direct histological and molecular data characterizing the CFFP remain limited. Nevertheless, its shared intra-articular location, synovial integration, and biomechanical exposure suggest a cellular architecture broadly similar to the IFP. By contrast, the IFP has been more extensively characterized as a heterogeneous and immunologically active intra-articular adipose tissue. In knee samples from 21 IFPs (6 healthy controls and 15 knee OA donors), recent single-nucleus and spatial transcriptomic work identified fibroblasts, macrophages, adipocytes, and endothelial cells as major IFP populations; OA stage was not uniformly reported across donors, and the distribution of these populations may vary by stage, obesity status, sex, and sampling context (Peters et al., 2025). Single-cell and transcriptomic studies further support extensive stromal and immune heterogeneity across the IFP and synovium, reinforcing the concept that these tissues function as a coordinated joint metabolic-immune unit (Peters et al., 2025; Tang et al., 2024). For the purposes of this review, we define “mechanical exposure” as the intermittent compressive and shear forces both fat pads experience within a moving synovial joint. Load magnitude, direction, and confinement differ between the patellofemoral IFP and the deep acetabular fossa, and direct comparative load bearing biomechanical data are lacking, so this is presented as a hypothesis. With this in mind, direct characterization of the hip fat pad is nonetheless emerging with studies profiling hip intra-articular adipose tissue (Eymard et al., 2017) and characterizing human pulvinar sub-synovial adipose tissue across aging and OA, reporting a disease-associated expansion of DPP4+/THY1+ mesenchymal stem/progenitor cells (Tilouche et al., 2026).

Fibroblasts, as the dominant stromal population, are likely central regulators of extracellular matrix turnover, inflammatory signaling, and communication with the synovium. Their abundance and functional relationship with synovial fibroblasts support the concept that the IFP and synovium act as an integrated joint metabolic-immune unit in OA (Wijesinghe et al., 2026; Peters et al., 2025; Tang et al., 2024). Macrophages, the second most abundant cell population, regulate the inflammatory state of the tissue and may adopt either pro-inflammatory (M1) or anti-inflammatory (M2) phenotypes in response to local signaling cues (Greif et al., 2020; Peters et al., 2025; Tang et al., 2024). In OA, pro-inflammatory macrophage activity is associated with mediators such as IL-6, TNF-α, and CTGF that promote catabolic and fibrotic changes (Greif et al., 2020; Zhang et al., 2020). In parallel, neuroinflammatory mediators such as SP and broader inflammatory cytokine signaling within the IFP/synovium complex further contribute to synovitis, fibrosis, and pain-associated pathology (Greif et al., 2020; Kouroupis et al., 2019; Liebmann et al., 2025). Adipocytes within the IFP function not only as lipid-storage cells but also as active endocrine and paracrine regulators of the joint microenvironment. These cells secrete adipokines and inflammatory mediators that influence synovial inflammation, cartilage metabolism, and immune cell recruitment, contributing to the tightly regulated communication network between the IFP, synovium, and cartilage that becomes dysregulated in OA (Greif et al., 2020; Tang et al., 2024). Lastly, endothelial cells form the vascular network of the IFP and support immune cell trafficking, nutrient delivery, and inflammatory signaling. Additional, less common, immune cells in the IFP include mast cells and memory T lymphocytes (both CD4 and CD8) further reinforcing its role as an immunologically responsive tissue capable of modulating both innate and adaptive immune responses (Ioan-Facsinay and Kloppenburg, 2013; Klein-Wieringa et al., 2016). Whether the CFFP harbors a comparable immune cell landscape remains an open question, but its synovial lining and vascularized stromal compartment suggest the structural prerequisites for similar immunological activity are present.

The cell population most relevant to the cell-based therapeutic framework described in this review is the IFP-derived MSC population, which has multilineage differentiation potential and immunomodulatory activity (Kouroupis et al., 2019; Kouroupis et al., 2020a; Kouroupis et al., 2020b). These cells have become a major focus of translational interest because they can regulate local inflammatory signaling and interact directly with neurogenic pathways. Additional studies from this group have shown that spheroid formation, extracellular vesicle (EV) profiling, and regulatory-compliant expansion strategies further support the therapeutic relevance of IFP-MSC in OA-associated inflammation and fibrosis (Kouroupis et al., 2021; Liebmann et al., 2024; Philippon et al., 2025; Kouroupis et al., 2022). The breadth of IFP-MSC characterization to date is summarized in Table 2. The consistency of canonical MSC immunophenotype markers (CD73, CD90, CD105 >95%) across multiple studies, alongside high CD10 expression linked to Substance P degradation, establishes IFP-MSC as the reference standard for joint-resident adipose-derived MSC therapy.

**TABLE 2:** IFP-MSC characterization data. Characterization findings for IFP-MSC as reported in the literature. This list is not exhaustive, however, these entries are grouped as MSC identity markers, functional readouts, and candidate EV-associated functional indicators. miRNA and immunomodulatory analytes are reported functional characteristics rather than identity markers. Future research should characterize CFFP-MSC and their relationship to IFP-MSC.

Assay	Marker/analyte	Finding	References
Flow cytometry	CD73, CD90, CD105, CD44	Positive (>95%)	Zhong et al. (2020), Gardashli et al. (2024), Han et al. (2021)
	CD34, CD45, CD56, HLA-DR	Negative (<2%)	Zhong et al. (2020), Gardashli et al. (2024), Han et al. (2021)
	CD10	24.0% (FBS)	Gardashli et al. (2024)
qPCR	IDO, HLA-G, IL-6, IL-10, CD10	Immunomodulatory	Kouroupis et al. (2019), Kouroupis et al. (2021)
	Oct-4, SOX2, NANOG, Nucleostemin	Stemness	Kouroupis et al. (2021), Radhakrishnan et al. (2019)
ELISA	PGE_2_, HLA-G, IL-6, IL-6 sR, IL-8, TNF-α, ICAM-1, IP-10, MCP-2, RANTES, IFN-γ, TIMP-2, sTNF-RII	Immunomodulatory cytokines	Kouroupis et al. (2019), Kouroupis et al. (2021), Kouroupis et al. (2020b), Garcia et al. (2016)
	AR, bFGF, bNGF, EGF, EGF R, FGF-4, GM-CSF, GDNF, HGF, IGFBP-2, IGFBP-4, IGFBP-6, IGF-1, IGF-1 sR, M-CSF, M-CSF R, NT-3, NT-4, PDGF-AA, PDGF Rα, SCF R, TGF-α, VEGF, VEGF R3, VEGF D	Growth factors	Kouroupis et al. (2019), Kouroupis et al. (2021), Kouroupis et al. (2020a)
miRNA (EV)	hsa-miR-142-3p, hsa-miR-146a, hsa-miR-107, hsa-miR-25-3p, hsa-miR-100-5p	Immune system regulation	Kouroupis et al. (2022), Wu et al. (2019), Li et al. (2022)
Protein (EV)	IL-6, IL-8, IP-10, TIMP-2, TGF-β2, VEGFD, βFGF, IGF-I sR	Immunomodulation and growth factors	Kouroupis et al. (2022)

In the hip, direct cellular characterization of CFFP-derived MSC remains more limited than that of IFP-MSC. What is currently established is narrower: the cotyloid fossa synovium contains MSC-like populations with self-renewal and differentiation potential (Murata et al., 2018). This supports the presence of biologically active stromal progenitors in the cotyloid fossa region, but it does not yet establish the phenotype or therapeutic function of directly isolated CFFP-MSC, work that has only begun (Tilouche et al., 2026). Based on the biological precedent established by IFP-MSC and given the shared synovial progenitor environment of the cotyloid fossa, we hypothesize that CFFP-MSC will demonstrate comparable immunomodulatory capacity, CD10 expression, and responsiveness to inflammatory priming, with hip-specific adaptations that may reflect the distinct mechanical and structural environment of the acetabulum. In contrast to the extensive dataset presented in Table 2 for IFP-MSC, no equivalent characterization exists for CFFP-MSC- no flow cytometry panel, no CFU-F quantification, and no secretome profiling has been reported directly from isolated CFFP tissue. Furthermore, donor characteristics influence fat-pad MSC biology. IFP cell composition and fibroblast phenotype vary with OA stage, sex, and obesity or BMI (Zeng et al., 2020; Peters et al., 2025), and MSC potency declines with donor age and inflammatory burden (Zhong et al., 2020; Garcia et al., 2016). Because reported CFFP samples derive largely from FAI or OA hips of differing age and sex, these donor factors should be matched or reported when comparing CFFP-MSC with IFP-MSC to avoid confounding tissue-of-origin differences.

## 4 Synovial composition and relationships

### 4.1 Synoviocytes

Both the CFFP and IFP are anatomically and functionally integrated with the synovial membrane, forming a morpho-functional unit (Slullitel et al., 2020; Eymard et al., 2017; Macchi et al., 2018). The synovium overlays the posterior aspect of the IFP while the synovium within the cotyloid fossa lines the non-articular surfaces of the acetabular fossa and envelops the ligamentum teres and CFFP (Slullitel et al., 2020; Zeng et al., 2020). In both tissues there is thought to be active molecular crosstalk, with the fat pads influencing synovial inflammation and *vice versa* (Eymard et al., 2017; Greif et al., 2020; Macchi et al., 2018; Faust et al., 2024).

For clarity, the synovial lining includes two broad synoviocyte populations: type A macrophage-like synoviocytes and type B fibroblast-like synoviocytes. Synoviocytes form the synovial lining, with type A cells functioning as macrophage-like phagocytes (CD68^+^) and type B cells serving as fibroblast-like synoviocytes (FLS) that produce hyaluronan, lubricin, and extracellular matrix (Greif et al., 2020; Emmi et al., 2022; Knab et al., 2022; Jay and Waller, 2014). This division is critical in OA as type A cells drive immune surveillance and debris clearance, while type B fibroblast-like synoviocytes become key mediators of synovial inflammation and fibrosis marked by podoplanin (PDPN), CD90, and α6β1 integrin (Greif et al., 2020; Emmi et al., 2022; Haubruck et al., 2021; Damerau et al., 2024; Pirilä et al., 2001). In OA, the synovium shifts toward invasive immune-effector subtypes, specifically CD90^+^/THY1^+^ fibroblast-like synoviocytes (FLS)- a pathogenic subpopulation of type B synoviocytes- along with differentiation into myofibroblast-like cells that promote fibrosis (Faust et al., 2024; Damerau et al., 2024). The cotyloid fossa synovium, which lines the non-articular acetabular surfaces and integrates directly with the CFFP, likely undergoes analogous fibroblast activation and synovial lining changes in hip OA; a hypothesis supported by the macrophage infiltration and cytokine upregulation recently documented in this region (Slullitel et al., 2020; Tilouche et al., 2026; Kuhns et al., 2025; Eymard et al., 2017).

Notably, lubricin (PRG4) which is secreted primarily by type B synoviocytes and, to a lesser extent, by chondrocytes and IFP-resident MSC, forms a protective boundary layer over articular cartilage that enables near-frictionless motion and shields chondrocytes from mechanical damage and apoptosis (Kouroupis et al., 2020a; Jay and Waller, 2014; Roggio et al., 2023; Chwastek et al., 2022). Its loss or degradation in OA disrupts joint lubrication, accelerates cartilage wear, and perpetuates the inflammatory cycle (Scanzello and Goldring, 2012; Jay and Waller, 2014; Roggio et al., 2023). We hypothesize that in the hip, type B synoviocytes and chondrocytes bear a greater share of lubricin production than the acetabular fat pad, representing a potentially important joint-specific difference in lubrication biology that warrants further investigation (Macchi et al., 2018; Jay and Waller, 2014). Given that the CFFP is anatomically integrated with the acetabular synovium, characterizing its contribution to lubricin production and boundary lubrication in the hip represents an important and currently unaddressed question, one that may have direct implications for understanding strategies for cartilage preservation in early hip OA.

### 4.2 MSC interactions in the joint space

Under physiological conditions the fat pads secrete adipokines with context-dependent actions, including adiponectin (predominantly anti-inflammatory) alongside resistin, visfatin, and leptin (predominantly pro-inflammatory) and FABP4 (Slullitel et al., 2020; Greif et al., 2020; Zeng et al., 2020; Liu et al., 2019; Sun et al., 2018; Gross et al., 2017), growth factors (basic fibroblast growth factor [bFGF], vascular endothelial growth factor [VEGF]), and bone morphogenetic protein 7 (BMP-7) (Ushiyama et al., 2003) that support synovial cell function, modulate immune cell activity, and contribute to the composition of synovial fluid via paracrine functions (Greif et al., 2020; Macchi et al., 2018; Faust et al., 2024). MSC from both tissues can differentiate into synovial fibroblasts or adipocytes, reflecting a shared progenitor pool and integrated tissue unit (Peters et al., 2025; Tang et al., 2024; Macchi et al., 2018). These MSC, shared between the fat pad and synovium contribute to tissue repair and renewal, while resident synovial macrophages and fibroblasts maintain barrier function and clear debris, preventing inflammation (Greif et al., 2020; Tang et al., 2024; Knab et al., 2022; Haubruck et al., 2021). The synovial fluid acts as a conduit for these mediators, facilitating bidirectional communication and nutrient exchange between the fat pad and synovium, further supporting joint lubrication and homeostasis (Greif et al., 2020; Ioan-Facsinay and Kloppenburg, 2013; Macchi et al., 2018). This integrated fat pad/synovium unit ensures that mechanical stress, metabolic demands, and immune surveillance are balanced, preventing chronic inflammation and maintaining joint function (Blackler et al., 2025; Macchi et al., 2018; Knab et al., 2022). By extension, if CFFP-MSC share the paracrine and adipokine-secreting capacity of IFP-MSC, as the shared adipose/synovial origin would suggest, they may contribute similarly to hip joint homeostasis, representing an untapped endogenous repair population whose disruption could actively participate in hip OA progression. This CFFP inference is drawn from IFP data and has not been demonstrated directly in the CFFP.

Disruption of these interactions, such as loss of adipocyte-derived glucocorticoid signaling or a shift toward pro-inflammatory cytokine production, leads to loss of homeostasis and initiation of joint pathology (Faust et al., 2024; Knab et al., 2022). Mechanistically, adipocytes in joint fat pads express high levels of 11β-hydroxysteroid dehydrogenase, which converts inactive cortisone to active cortisol locally (Faust et al., 2024). This local cortisol then signals through the glucocorticoid receptor (GR) in neighboring synovial fibroblasts, driving a healthy phenotype characterized by enhanced fatty acid metabolism, lipid transport, and suppression of pro-fibrotic and pro-inflammatory pathways (Faust et al., 2024). Loss of adipocytes or disruption of the GR signaling in fibroblasts leads to loss of the healthy fibroblast state, increased matrix remodeling, and, for GR disruption, worsened joint disease in a mouse inflammatory-arthritis model (Faust et al., 2024). Whether this axis governs fibroblast homeostasis in OA specifically has not been established in the literature to this point. In OA-specific models, disrupting endogenous glucocorticoid signaling in chondrocytes or osteoblasts attenuates rather than accelerates cartilage damage (Macfarlane et al., 2023; Tu et al., 2019), and endogenous glucocorticoids are anti-inflammatory in rheumatoid arthritis yet detrimental in OA (Macfarlane et al., 2024). The role of local glucocorticoid signaling in the fat pad-synovium unit is therefore disease-context-dependent. In addition to these regulatory mechanisms, a specialized population of PRG4^+^ superficial zone chondrocytes serve as an additional major source of lubricin; loss or dysfunction of these cells is independently associated with early cartilage degeneration and OA progression (Liebmann et al., 2024; Jay and Waller, 2014; Roggio et al., 2023).

### 4.3 A context dependent phenotype of the fat pad-synovium unit

The molecular profiles of knee and hip fat pad-synovium units are context-dependent, meaning their secretory output shifts between anti-inflammatory and pro-inflammatory phenotypes depending on the local joint environment, disease state, and nature of the inflammatory stimulus. In regard to an anti-inflammatory environment, the main mediators released by the IFP include adiponectin, IL-10, and TGF-β (Greif et al., 2020; Ioan-Facsinay and Kloppenburg, 2013; Zeng et al., 2020; Bastiaansen-Jenniskens et al., 2013). These mediators are produced mainly by adipocytes, MSC, and M2 macrophages (Greif et al., 2020; Kouroupis et al., 2021; Bastiaansen-Jenniskens et al., 2013). Both adiponectin and adipsin are secreted at higher levels by the IFP compared to subcutaneous adipose tissue, and their actions collectively dampen inflammatory cascades, reduce catabolic enzyme activity, and support joint homeostasis in OA (Ioan-Facsinay and Kloppenburg, 2013; Gross et al., 2017).

Adiponectin, in particular, is consistently elevated in the IFP compared to subcutaneous adipose tissue and is associated with immunomodulatory and anti-inflammatory effects, including inhibition of pro-inflammatory cytokine production and reduction of MMP activity (Ioan-Facsinay and Kloppenburg, 2013; Gross et al., 2017; Ilia et al., 2022). Adiponectin exerts anti-inflammatory effects in the joint by binding to its receptors (AdipoR1 and AdipoR2) on synovial cells, chondrocytes, and macrophages, leading to activation of AMPK and PPAR-α signaling (Ilia et al., 2022; Koskinen et al., 2011; Kang et al., 2010; Chinetti et al., 2004). In some settings, adiponectin has been reported to reduce pro-inflammatory cytokine production (such as TNF-α and IL-1β) and MMP expression in chondrocytes and synoviocytes (Chen et al., 2006; Lee et al., 2008). Adiponectin also promotes the release of anti-inflammatory cytokines like IL-10, perhaps mitigating IL-6 mediated effects, which could further dampen inflammatory cascades in the joint microenvironment (Wolf et al., 2004; Folco et al., 2009). Adiponectin’s actions are nonetheless context dependent. Although predominantly anti-inflammatory in homeostasis, it is elevated in OA and rheumatoid synovial fluid and can- again depending upon the context- promote IL-6, IL-8, and MMP production, so its net effect depends on receptor context, isoform, and disease stage (Zeng et al., 2020; Koskinen et al., 2011; Kang et al., 2010; Tong et al., 2011).

Adipsin (also known as complement factor D) is a serine protease involved in the alternative complement pathway (Rosen et al., 1989; White et al., 1992). In the IFP and CFFP, we hypothesize that locally generated complement fragments could favor anti-inflammatory macrophage phenotypes and dampen pro-inflammatory cytokine release, indirectly limiting MMP activity and cartilage degradation; however, this specific sequence has not been demonstrated in fat-pad tissue and remains speculative.

Although direct molecular profiling of the CFFP under homeostatic conditions is lacking, its shared adipose-synovial composition and intra-articular location provide a mechanistic basis to hypothesize that it produces a broadly similar anti-inflammatory secretome, including adiponectin and adipsin. If true, these factors could contribute to hip joint homeostasis and would likely also become dysregulated in disease. Table 3 synthesizes this evidence gap systematically across key translational categories. The asymmetry is striking for nearly every domain, where the IFP column reflects an established and growing evidence base, while the CFFP column reflects absence or inference.

**TABLE 3:** Evidence and translational readiness of IFP vs. CFFP in OA anti-inflammatory phenotype. Readiness distinguishes preclinical evidence maturity from clinical readiness. IFP-based therapies remain in early stages in terms of clinical readiness with only small human series and no large scale randomized controlled trials.

Category	IFP (knee)	CFFP (hip)
Tissue definition	Well-defined anatomical and functional IFP/synovium unit (Emmi et al., 2022; Macchi et al., 2018)	Fibrofatty tissue within the cotyloid fossa; less consistently studied as a functional unit (Slullitel et al., 2020; Murata et al., 2018)
MSC isolation and potency markers	Robust isolation with chondrogenic and immunomodulatory potential; CD10/neprilysin identified as functional potency marker (Greif et al., 2020; Kouroupis et al., 2019; Kouroupis et al., 2020a; Sun et al., 2018; Tangchitphisut et al., 2016)	Cotyloid fossa synovium MSC isolated with high self-renewal and differentiation potential; no CFFP-MSC potency marker characterization reported (Tilouche et al., 2026; Murata et al., 2018)
Inflammatory mediators	Strong evidence for IL-6, TNF-α, and adipose-derived mediators in OA pathogenesis (Ioan-Facsinay and Kloppenburg, 2013; Kapoor et al., 2011; Zhang et al., 2020; Wijesinghe et al., 2026)	Macrophage infiltration and cytokine upregulation documented in the pulvinar region; specific mediator profiling limited (Kuhns et al., 2025; Eymard et al., 2017)
Neurogenic mediators	SP and CGRP contribute to IFP-driven inflammation and pain signaling (Liebmann et al., 2025; Zeng et al., 2020; Liebmann et al., 2024)	Not functionally characterized in the CFFP; inferred from cotyloid fossa synovial studies (Tilouche et al., 2026; Saxler et al., 2007)
Priming and EV evidence	MSC function enhanced by inflammatory, hypoxic, and hormonal priming; EVs demonstrate anti-inflammatory and regenerative potential in OA (Philippon et al., 2025; Zhong et al., 2020; Gardashli et al., 2024; Wu et al., 2019; Moeinabadi-Bidgoli et al., 2023)	No priming or EV characterization reported
Clinical translation	Preclinical and early translational evidence supports immunomodulation and cartilage regeneration; large-scale RCTs lacking	Primarily observational; no clinical translation studies reported
Overall readiness	High: well-characterized mechanistic and translational foundation	Limited: biologically plausible but lacking direct characterization and mechanistic depth

### 4.4 The pro-inflammatory/profibrotic phenotype

In OA, the synovium plays a central role in initiating and amplifying joint inflammation through the production of pro-inflammatory cytokines, including IL-1β, TNF-α, IL-6, IL-8, and monocyte chemoattractant protein-1 (MCP-1) (Katz et al., 2021; Bondeson et al., 2006; Scanzello and Goldring, 2012; Zhang et al., 2020; Chou et al., 2020). These mediators are primarily secreted by synovial macrophages and fibroblast-like synoviocytes (FLS) and contribute to synovial lining hyperplasia, immune cell recruitment, and cartilage degradation. The synovium further amplifies inflammation through continued cytokine production, which is upregulated in response to fat pad-derived inflammatory signals such as IL-6, IL-8, and PGE2, establishing a positive inflammatory feedback loop between the synovium and fat pad (Eymard et al., 2017; Zeng et al., 2020; Bondeson et al., 2006; Scanzello and Goldring, 2012).

IFN-γ and TNF-α are primarily secreted by activated CD4^+^ T cells (particularly Th1 cells) and macrophages, with chondrocytes also contributing to TNF-α production under inflammatory conditions (Robinson et al., 2016; Klein-Wieringa et al., 2016; Ishii et al., 2002; Rosshirt et al., 2019). In OA, synovial fluid and synovial membrane samples are enriched in activated Th1 cells compared to peripheral blood, contributing to elevated IFN-γ levels (Klein-Wieringa et al., 2016; Ishii et al., 2002; Rosshirt et al., 2019; Rosshirt et al., 2021). CD14^+^ macrophages predominate across OA samples and, when polarized toward the M1 phenotype, intensify inflammation through TNF-α and, to a lesser extent, IFN-γ release (Zhang et al., 2020; Klein-Wieringa et al., 2016; Kulkarni et al., 2022; Moradi et al., 2015). The OA synovial fluid microenvironment further reinforces this M1 polarization, sustaining elevated production of TNF-α and IFN-γ (Kulkarni et al., 2022).

In pathological states, these inflammatory cascades extend to the fat pads, which adopt a pro-inflammatory and profibrotic phenotype. The IFP releases elevated levels of pro-inflammatory cytokines (IL-6, IL-8, IFN-γ, TNF-α), chemokines (MCP-1), PGE2, and MMPs, further promoting synovial inflammation, synovial lining hyperplasia, and immune cell infiltration (Eymard et al., 2017; Greif et al., 2020; Wijesinghe et al., 2026; Haubruck et al., 2021).

The catabolic enzymes produced by the synovial tissue in this context include matrix metalloproteinases (MMP-1, MMP-3, MMP-9, MMP-13), aggrecanases (ADAMTS-4, ADAMTS-5, ADAMTS-7, ADAMTS-12), cathepsin K, and cathepsin S (Bondeson et al., 2006; Chwastek et al., 2022; Estell et al., 2019; Pérez-García et al., 2016). Cathepsin K, a lysosomal protease associated with collagen degradation, has been implicated in OA progression (Mijanović et al., 2022). Cathepsin S, expressed primarily in antigen-presenting cells, contributes to chronic inflammatory processes and immune dysregulation (Gao et al., 2025). Together, these enzymes mediate extracellular matrix breakdown and progressive joint damage.

Within the OA knee, effects on the IFP have been extensively characterized, demonstrating elevated levels of IL-6, VEGF, leptin, prostaglandin F2α (PGF2α), collagen cross-linking enzymes, and visfatin (Eymard et al., 2017; Zeng et al., 2020; Bastiaansen-Jenniskens et al., 2013). This shift from a homeostatic to a pro-inflammatory and profibrotic phenotype promotes angiogenesis, macrophage infiltration, and synovial fibrosis. Adipokines such as leptin correlate with OA severity and cartilage degeneration, while fibrotic mediators including PGF2α and collagen cross-linking enzymes contribute to joint stiffness and pain through extracellular matrix remodeling (Robinson et al., 2016; Ioan-Facsinay and Kloppenburg, 2013; Emmi et al., 2022; Bastiaansen-Jenniskens et al., 2013).

In contrast, the inflammatory and molecular profile of the CFFP remains far less well characterized. Available evidence suggests that key inflammatory mediators, including IL-6, IL-8, and PGE2, are likely shared between the IFP and CFFP (Tilouche et al., 2026; Eymard et al., 2017); however, direct molecular profiling of the CFFP remains limited. Much of the existing evidence is derived from studies of intra-articular adipose tissue or pulvinar synovium rather than true fat pad-specific analyses. While it is plausible that the CFFP participates in hip joint inflammation through similar IL-6 and VEGF-mediated pathways, this remains speculative and unvalidated experimentally. This highlights a critical gap in musculoskeletal biology and underscores the need for targeted characterization of CFFP-specific cellular and molecular profiles. A working mechanistic framework, supported by the IFP literature, would predict that the CFFP, under pathological conditions, upregulates a comparable pro-inflammatory and profibrotic program involving IL-6, IL-1β, MMP-13, and ADAMTS-5, with the confined area of the acetabular fossa potentially amplifying the local consequences of this signaling. Directly testing this prediction is a primary objective of our ongoing work.

## 5 IFP/synovium and cotyloid fossa synovium as a source of MSC for cell therapy

IFP-MSC have been extensively studied due to their accessibility during routine knee procedures and their robust proliferative capacity. They demonstrate chondrogenic differentiation potential, immunomodulatory properties, and may be less susceptible to age- and inflammation-related functional decline than other MSC sources (Greif et al., 2020; Sun et al., 2018; Liao et al., 2022; Vahedi et al., 2021). Intra-articular administration of IFP-MSC products has been reported as safe, with preliminary evidence of improvement in pain and functional outcomes in knee OA patients (Chen et al., 2022). Early imaging findings further suggest potential cartilage-protective effects, though larger controlled trials are required to confirm efficacy (Greif et al., 2020; Vahedi et al., 2021; Chen et al., 2022; Carneiro et al., 2023). Priming of IFP-MSC further enhances their immunomodulatory and chondroprotective effects, as demonstrated by increased anti-inflammatory cytokine production and chondroprotective activity *in vitro* (Kouroupis et al., 2019; Philippon et al., 2025; Gardashli et al., 2024), with cartilage protection and improved gait reported in an animal OA model (Wu et al., 2024).

The promise that MSC-EVs will serve to be therapeutic must be considered in the context that there is evidence showing that EV effects can be heterogeneous. A recent study showed that a mitochondria-derived EV subpopulation from IFP-MSC transferred mitochondrial DNA to chondrocytes, activated cGAS-STING signaling, and worsened cartilage degradation and synovitis in a rat OA model, with STING inhibition reversing these effects (Li et al., 2026). Donor disease state, EV subtype, and cargo composition influenced how harmful the EV preparation was (Li et al., 2026); aspects specifically related to our discussion. The pathogenic activity was attributed to a mitochondria-derived vesicle subpopulation that was enriched in OA joints (Li et al., 2026), so a large portion of CFFP-EV preparation could carry harmful cargo that whole-population characterization would miss. This implies that subpopulation-level profiling and fractionation, rather than bulk isolation, will be required before any CFFP-EV therapeutic.

### 5.1 MSC immunomodulation: polarization dynamics and regulation of macrophage and T-cell responses

MSC exhibit functional plasticity that is strongly influenced by their surrounding microenvironment, giving rise to two broad functional phenotypes often referred to as MSC1 (pro-inflammatory) and MSC2 (anti-inflammatory) (Kouroupis et al., 2019; Waterman et al., 2010). Exposure to pathogen-associated molecular patterns such as lipopolysaccharide (LPS) through Toll-like receptor 4 (TLR4) promotes the MSC1 phenotype, which is associated with increased secretion of pro-inflammatory mediators including IL-6 and TNF-α, thereby supporting immune activation (Waterman et al., 2010; Noronha et al., 2019). In contrast, stimulation through Toll-like receptor 3 (TLR3), such as double-stranded RNA, or exposure to inflammatory cytokines including IFN-γ and TNF-α, drives MSC toward the MSC2 phenotype, characterized by enhanced immunosuppressive activity and secretion of regulatory factors such as IL-10, PGE2, and tumor necrosis factor-stimulated gene-6 (TSG-6) (Waterman et al., 2010; Le Blanc and Mougiakakos, 2012; Deng et al., 2020).

The principal immunoregulatory role of MSC lies in their interaction with macrophages and T cells. Activated MSC promote a shift from M1 to M2 macrophages through PGE2-dependent mechanisms and TSG-6 secretion, resulting in decreased production of TNF-α and IL-12 and increased expression of IL-10, CD206, and CD163, markers associated with tissue repair and resolution of inflammation (Le Blanc and Mougiakakos, 2012; Prock and op, 2013; Kim and Hematti, 2009; Melief et al., 2013). This macrophage reprogramming reduces oxidative stress, suppresses NF-κB signaling, and limits further inflammatory damage within the tissue microenvironment (Prock and op, 2013; Oh et al., 2014).

MSC also exert significant control over T cell responses. MSC2-polarized cells inhibit T cell proliferation, suppress effector T helper cell activity, and promote the expansion of regulatory T cells (Tregs), contributing to immune tolerance and attenuation of chronic inflammation (Waterman et al., 2010; Le Blanc and Mougiakakos, 2012; Qiu et al., 2017). This immunosuppressive effect is mediated through the release of soluble factors such as indoleamine 2,3-dioxygenase, IL-10, and PGE2, which interfere with T cell activation and cytokine secretion (Kouroupis et al., 2021; Waterman et al., 2010; Le Blanc and Mougiakakos, 2012; Aggarwal and Pittenger, 2005). Through these coordinated effects on both innate and adaptive immune cells, MSC play a central role in shaping local inflammatory states and determining whether the environment favors tissue degeneration or repair.

Cell-free therapies, using MSC-EVs are particularly promising for their immunomodulatory and chondroprotective effects, with potential for engineered personalized and targeted treatment. EVs deliver bioactive cargo (miRNAs, proteins, lipids) that directly enhance chondrocyte proliferation, inhibit apoptosis, and stimulate extracellular matrix synthesis, thereby protecting and regenerating cartilage (Kouroupis et al., 2022; Liao et al., 2022; Wu et al., 2019). As discussed above, MSC-EVs modulate the joint microenvironment as part of the paracrine signaling effect- shifting macrophages towards M2, suppressing pro-inflammatory cytokines stimulating the release of anti-inflammatory cytokines. The effect of this signaling leads to reduced synovial inflammation and supports cartilage homeostasis and repair (Kouroupis et al., 2022; Liao et al., 2022; Wu et al., 2019).

### 5.2 MSC primed re-injection

Priming MSC prior to re-injection enhances immunomodulatory potency and therapeutic effect (Kouroupis et al., 2019; Gardashli et al., 2024; Moeinabadi-Bidgoli et al., 2023). Clinical translation of MSC therapy is constrained by poor post-transplant survival, inefficient homing, and reduced potency within inflammatory joint environments. Three complementary preconditioning strategies have been developed to address this: hypoxic culture, cytokine-based inflammatory priming, and hormonal priming with oxytocin (OXT) (Gardashli et al., 2024; Moeinabadi-Bidgoli et al., 2023; Kay et al., 2021).

Hypoxic preconditioning (3% O_2_) underpins the marrow-isolated adult multilineage inducible (MIAMI; bone-marrow-derived) platform, which generates a homogeneous, developmentally immature MSC subpopulation with sustained stemness across >30 population doublings and markedly reduced senescence compared to normoxic counterparts (Ley et al., 2026; D’Ippolito et al., 2004; Rios et al., 2016). Cytokine-based inflammatory priming with IFN-γ ± TNF-α/IL-1β drives MSC toward an immunosuppressive MSC2 phenotype, upregulating IDO, HLA-G, PD-L1, PGE_2_, and TSG-6 and promoting regulatory T cell expansion (Gardashli et al., 2024; Noronha et al., 2019; Le Blanc and Mougiakakos, 2012; Deng et al., 2020). In a MSC-macrophages co-culture system, OXT with inflammation primed MSC shifted the co-cultured macrophages transcriptional response, upregulating TLR1 19-fold and STAT1/TGFB1 >30-fold and promoting M2 polarization compared with inflammatory priming alone (Gardashli et al., 2024). This framework has been further expanded through Adeno-Associated Virus (AAV)-mediated transduction of a CGRP antagonist gene (aCGRP), generating an engineered IFP-MSC subpopulation with enhanced analgesic and anti-inflammatory EV cargo targeting neurogenic pain pathways in OA (Liebmann et al., 2025; Liebmann et al., 2024).

Figure 2 depicts the convergence of these three priming axes (hypoxic, cytokine-based, and hormonal) and their downstream immunomodulatory outputs. The key translational insight illustrated is not that anyone strategy is sufficient, but that combining these strategies is proposed to generate an MSC phenotype with simultaneously enhanced immunosuppressive, anti-fibrotic, and analgesic capacity. This is particularly relevant considering, to our knowledge, the three-way combination has not been tested directly.

**FIGURE 2:**
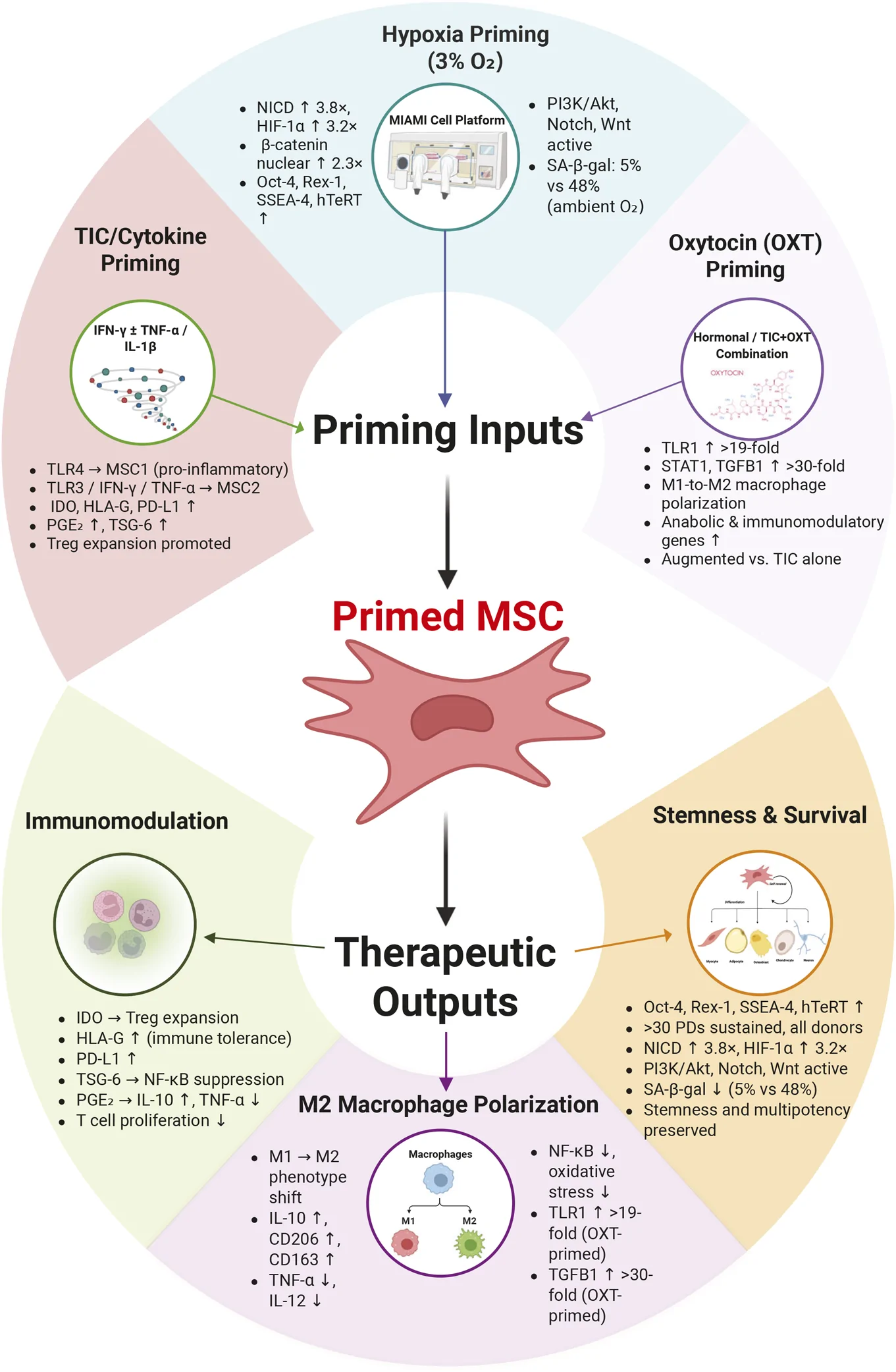
Overview of MSC priming strategies and downstream therapeutic outputs. The schematic illustrates three complementary preconditioning approaches applied to MSC prior to therapeutic re-injection. The hypoxic (MIAMI) findings derive from marrow-isolated adult multilineage inducible cells (bone marrow); cytokine and oxytocin priming findings derive from IFP-MSC. Top: Hypoxic priming (3% O_2_; MIAMI platform) activates PI3K/Akt, Notch, and Wnt signaling, sustaining stemness across >30 population doublings and reducing senescence from 48% to 5% versus ambient oxygen. Left: Cytokine-based inflammatory priming with IFN-γ ± TNF-α/IL-1β drives MSC from a pro-inflammatory MSC1 phenotype toward an immunosuppressive MSC2 phenotype, upregulating key immunomodulatory effectors including IDO, HLA-G, PD-L1, PGE_2_, and TSG-6 and promoting Treg expansion. Right: Hormonal priming with OXT in combination with inflammatory priming further augments immunomodulatory gene expression compared to TIC alone, upregulating in macrophages TLR1 19-fold and STAT1/TGFB1 more than 30-fold, while robustly promoting M1-to-M2 macrophage polarization and anabolic gene programs. TLR1 and STAT1/TGFB1 fold-changes were measured in co-cultured macrophages, not in the primed MSC directly. Center: Convergence of these three priming axes generates a primed IFP-MSC with an enhanced immunomodulatory phenotype. Bottom: Downstream therapeutic outputs include immunosuppression via IDO-mediated Treg expansion, HLA-G and PD-L1 upregulation, TSG-6-driven NF-κB suppression, and PGE_2_-induced IL-10 elevation with concurrent TNF-α reduction; M2 macrophage polarization characterized by increased IL-10, CD206, and CD163 and decreased TNF-α, IL-12, NF-κB activity, and oxidative stress; and preservation of MSC stemness and multipotency across more than 30 population doublings. Abbreviations: IDO, indoleamine 2,3-dioxygenase; MIAMI, marrow-isolated adult multilineage inducible; NICD, Notch intracellular domain; OXT, oxytocin; PD, population doubling; PD-L1, programmed death-ligand 1; PGE_2_, prostaglandin E_2_; SA-β-gal, senescence-associated β-galactosidase; SSEA-4, stage-specific embryonic antigen-4; STAT1, signal transducer and activator of transcription 1; TGFB1, transforming growth factor beta 1; Toll-like receptor inflammatory cocktail (TIC); TLR, toll-like receptor; Treg, regulatory T cell; TSG-6, tumor necrosis factor-stimulated gene-6. Created in BioRender by Rossow, R.

## 6 Future research into CFFP-MSC

IFP-MSC already provide a proof-of-concept for a theoretical model using CFFP-MSC as a potential treatment for hip OA (Kouroupis et al., 2019; Chen et al., 2022). IFP-MSC are well adapted to the knee joint environment, displaying robust chondrogenic potential, high immunomodulatory potency, and resilience to inflammatory stress (Kouroupis et al., 2019; Zhong et al., 2020; Liao et al., 2022), yet the hip remains an unresolved frontier. Despite the CFFP forming an anatomically parallel synovium-fat-MSC unit as the IFP, the biology of CFFP-MSC is still in its infancy. No studies have defined their immunomodulatory capabilities, differentiation potential, or ability to respond to inflammatory priming, nor compared them to other MSC types.

Despite hip OA affecting millions of people and being one of the most disabling musculoskeletal conditions worldwide, no validated disease-modifying orthobiologic therapy exists (Katz et al., 2021; Klop et al., 2025; Cross et al., 2014). As Table 3 makes clear, the translational readiness gap between IFP and CFFP spans every level from basic characterization to clinical application. This gap carries major therapeutic implications. If CFFP-MSC behave slightly differently from IFP-MSC, as is likely given the distinct mechanical, inflammatory, and structural environments of the hip, then current MSC therapies derived from bone marrow or abdominal adipose tissue may be suboptimal for hip OA. Identifying a hip-specific stromal population with strong anti-inflammatory and reparative capacity could fundamentally change how hip disease is treated. This potential advantage of hip-resident cells remains a testable hypothesis and does not imply inferiority of other MSC sources; the current evidence does not establish superiority.

## 7 Conclusion

The IFP provides a strong biological and translational reference model for studying joint-resident adipose-synovial MSC in OA. The cotyloid fossa/pulvinar region shares several structural features with the IFP, including intra-articular location, synovial integration, and fibrofatty composition, but its MSC and EV biology remains insufficiently characterized. The central conclusion of this review is therefore not that CFFP-MSC are equivalent to IFP-MSC, but that this comparison is scientifically testable and clinically relevant.

The most immediate need is baseline characterization of CFFP-MSC via surface phenotyping, clonogenic capacity, multilineage potential, and basal immunomodulatory gene expression. Findings will allow the scientific community to determine whether the cotyloid fossa harbors a MSC population functionally comparable to IFP-MSC. The second priority is functional comparison under controlled inflammatory stimuli, applying cytokine, oxytocin, and hypoxia priming paradigms now established for IFP-MSC to test whether CFFP-MSC respond with equivalent induction of IDO, HLA-G, PD-L1, PGE_2_, and TSG-6, and whether they drive comparable M2 macrophage polarization and Treg expansion. The comparative biology described here must precede therapeutic development, not accompany it. Until that foundation is established, CFFP-MSC and CFFP-EVs cannot be presented as validated hip-specific orthobiologic candidates.

Hip OA disproportionately affects working-age adults, and its disease-modifying therapeutic pipeline trails that of the knee by a substantial margin. The growing recognition that early hip OA involves synovial inflammation, fat pad activation, and neurogenic pain signaling means the window for biologic intervention is earlier than previously appreciated. If CFFP-MSC carry the immunomodulatory signatures of IFP-MSC they would represent an endogenous, arthroscopically accessible, and disease-relevant therapeutic source for the patients most in need of early joint-preserving treatment. Resolving the IFP/CFFP comparison is a prerequisite for extending the biology of joint-resident MSC therapy from the knee, where it is maturing, to the hip, where it has not yet begun.
